# Direct oral anticoagulants versus warfarin: is new always better than the old?

**DOI:** 10.1136/openhrt-2017-000712

**Published:** 2018-02-07

**Authors:** John Burn, Munir Pirmohamed

**Affiliations:** 1 Institute of Genetic Medicine, Newcastle Univerisity, Centre for Life, Newcastle upon Tyne, UK; 2 Department of Molecular & Clinical Pharmacology, Institute of Translational Medicine, University of Liverpool, Liverpool, UK

**Keywords:** stroke, warfarin, anticoagulants, atrial fibrillation

## Abstract

About 1.4 British million people are at risk of strokes due to non-valvular atrial fibrillation (AF) necessitating long-term anticoagulation. The vitamin K antagonist, warfarin, has a long half-life and narrow therapeutic range necessitating regular monitoring and is a common cause of iatrogenic hospital admission. Direct-acting oral anticoagulants (DOACs), dabigatran, rivaroxaban, apixaban and edoxaban are not required to have monitoring but are sensitive to changes in renal function and are associated with poorer adherence. There are good grounds to believe that DOACs are not always superior to warfarin in routine practice particularly with an older population. Much higher levels of therapeutic effectiveness can be achieved using a simple genotype guidance to identify those who are highly sensitive and by adoption of home monitoring. These adjustments could make warfarin the preferred drug for most people and would reduce the dramatic rise in health service expenditure.

It is estimated that 1.4 British million people are at risk of strokes due to non-valvular atrial fibrillation (AF) including about 4% of the over 80s.^1^ Antiplatelet agents are ineffective necessitating long-term anticoagulation.^2 3^ The *S*-isoform of the current standard, warfarin, works by interfering with vitamin K activation of clotting factors II, VII, IX and X.^4^ Its long half-life and narrow therapeutic range necessitate regular monitoring using the international normalised ratio (INR) which needs to be held in a range of 2–3 to minimise clot formation or the converse, uncontrolled bleeding.^5–7^ The latter is one of the most common causes of iatrogenic hospital admission.^8^

Two strategies have emerged to improve the clinical utility of preventive anticoagulation: (a) the development of a new class of anticoagulants, known as the direct-acting oral anticoagulants (DOACs), including dabigatran, rivaroxaban, apixaban and edoxaban; and (b) better optimisation of warfarin use by understanding the common genetic variation in drug response and by making INR testing and dose adjustment more convenient with self-testing devices.

A series of large-scale randomised controlled trials (RCTs) published over the last five years have demonstrated apparent superiority of DOACs over warfarin for key indicators coupled with the advantage of a wider therapeutic range and a lack of a need for regular monitoring of the degree of anticoagulation.^9–11^ This resulted in the UK National Institute for Health and Care Excellence (NICE) guidance in 2014 that the approaches should be given equal weight at patient initiation consultations (http://nice.org.uk/guidance/cg180) and subsequently a more direct endorsement of DOACs by the European Society of Cardiology.^12^ As a result, there is a major shift towards DOACs in clinical practice which is placing a significant burden on health budgets. In the National Health Service (NHS) in England, expenditure on anticoagulants rose by >£100 million in 2015/2016 and by around £400 million in the last financial year^i^. On present trends, the cost will rise to £1 billion per year by 2020, representing 5% of the total drug budget. While the price will begin to fall in 2022 as patents expire, these costs justify a critical examination of the available evidence.

The first DOAC to be licensed, dabigatran, is a thrombin inhibitor. Some degree of interpersonal variation in response was not made clear in initial submissions leading to a prolonged legal challenge.^13^ A reversal agent, idarucizumab, is now licensed in the UK but is very expensive, costing >£2500 per use as opposed to parenteral vitamin K as a reversal agent for warfarin.^14^ The other three licensed agents—rivaroxaban, apixaban and edoxaban—are direct inhibitors of activated factor X. Andexanet alfa, a reversal agent of factor Xa inhibitor activity, is under consideration to be licensed in the UK though its short half-life and high cost of >£1500 is an issue.^15^ The most frequently prescribed anti-Xa inhibitor, rivaroxaban, was shown to be non-inferior to warfarin in a large-scale trial which used a device for INR monitoring in the warfarin arm which was subsequently withdrawn on the US Food and Drug Administration instruction as it was unreliable.^16^ The original study authors concluded this had not compromised the final conclusions.

A meta-analysis of the four RCTs comparing efficacy of DOACs versus warfarin found the DOACs to be superior to warfarin in terms of embolic and haemorrhagic stroke but to be inferior in terms of gastrointestinal bleeding events.^17^ An important qualification was that superiority was dependent on the proportion of warfarin patients in the therapeutic window. If the average time in the therapeutic range (TTR) was <66%, the new drugs were superior but they were not superior if average TTR of patients on warfarin was >66% (Relative Risk =0.69, 95% CI 0.59 to 0.81 vs Relative Risk=0.93, 95% CI 0.76 to 1.13, P_interaction_=0.022).

Three other subsequent observations raise major concerns. RCTs tend to focus on younger people with fewer comorbidities. Abrahams *et al*^18^ reported in 2015 that adverse events were more common in the older population. This has a plausible biological basis since excretion rates are pivotal to maintaining correct therapeutic levels. DOACs are more dangerous in people with impaired renal function. In the frail elderly population, intercurrent illness can lead to acute decline in renal function leading to excessive anticoagulation and sometimes life-threatening bleeding. Guidance suggests that renal function should be regularly checked but compliance with this guidance is thought to be poor in practice. The importance of drug–drug interactions with DOACs is underappreciated and can occur with drugs used intermittently, for example, antibiotics such as clarithromycin. The lack of validated laboratory tests to monitor the degree of anticoagulation with DOACs means that any dose reduction in patients with impaired renal function or on interacting drugs is empirical.

The second major concern is adherence. A recent analysis (figure 1) has revealed much poorer adherence for the DOACs probably due to the lack of routine monitoring and in the case of dabigatran and apixaban, the need for twice-daily use. This analysis of adherence in England is based on a representative review of repeat prescription issuance and reveals a worrying decline in correct use over the first 12 months to just over half for rivaroxaban and apixaban and to 34% for dabigatran. These levels compare unfavourably with the 74% adherence for warfarin. Similar results have been reported in Canada.^19^ When the different age profile of patients in clinical practice and these new data on adherence are factored in, it becomes less likely that the DOACs will be superior to warfarin and are likely to be inferior. In those centres where TTR is >70%, these drugs are almost certainly inferior in routine practice. It is noteworthy that there is currently no systematic assessment of warfarin monitoring services. This would allow commissioners to make a more objective assessment of the current trend towards DOACs as treatment of first choice.

**Figure 1:**
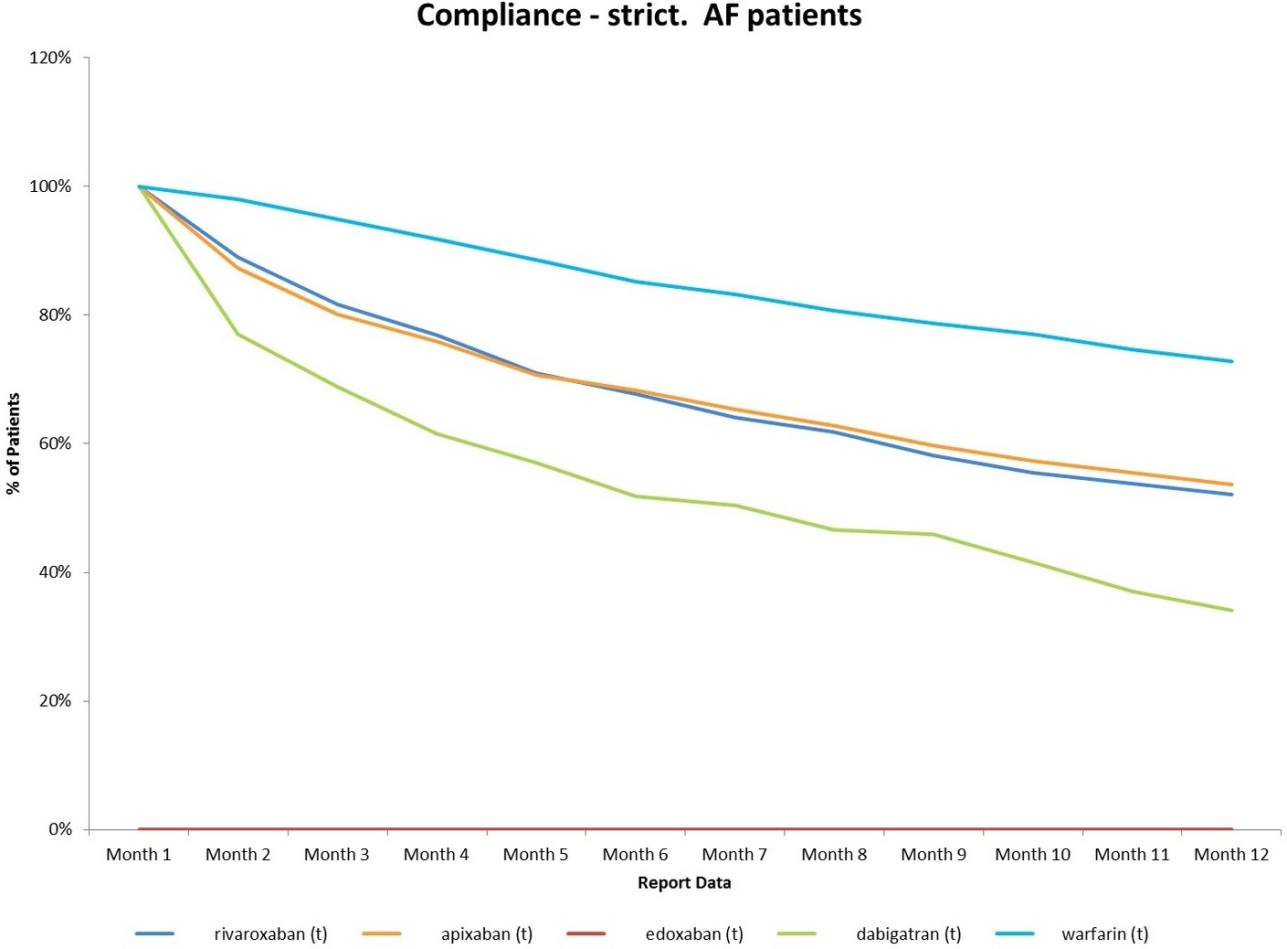
Adherence data for oral anticoagulants in 380 nationally distributed general practices (2143 general practitioners) in September 2016 using selected InPS Vision clinical systems. AF, atrial fibrillation.

The third area of concern is the association between the use of DOACs and myocardial infarction. This was initially identified with dabigatran and has been confirmed by meta-analysis.^20^ Although initially dismissed as due to a protective effect of warfarin, rather than an adverse effect of dabigatran, a recent mechanistic study has shown that dabigatran increases platelet adhesion and aggregation.^21^ Worryingly, more recently, it has been suggested in an observational study,^22^ and in a meta-analysis of randomised trials, that the anti-Xa DOACs may also increase the risk of myocardial infarction.^23^ Further studies are needed in this area to reduce any confounders and should include a mechanistic evaluation.

A possible tipping point in the debate has emerged recently; a systematic review of warfarin use and cancer rates using comprehensive Norwegian population-based ascertainment involving >1.2 million people has revealed a significant reduction in common cancers among warfarin users (Incident Rate Ratio 0.84, CI 0.82 to 0.86).^24^ Secondary analysis focused on those with AF revealed an incidence rate ratio of 0.62 (CI 0.59 to 0.65) with a relative risk of only 0.39 for lung cancer. In vitro studies support an anticancer effect, possibly mediated via the vitamin K-dependent product of the gene Gas6, the ligand for Axl receptor tyrosine kinase. If this observation is confirmed, the case for preferring warfarin, especially in the older population, becomes convincing.

There have been two major developments which make it possible to further improve the clinical outcomes in patients on warfarin. It has been recognised for 20 years that approximately 34% of the population carry one of two variants, known as *2 (rs1799853) and *3 (rs1057910), in the *CYP2C9* gene which is responsible for breakdown of the more active S-form of warfarin.^25^ People who are homozygous for either or doubly heterozygous have a much slower decline in warfarin levels and require much lower maintenance doses. Stability is harder to achieve and TTR is reduced. A second gene, *VKORC1*, codes for the enzyme targeted by warfarin. A common variant c.1639 G >A(rs9923231), present in 58% of the population, reduces the expression of this gene and hence reduced levels of the enzyme, making the coagulation system more sensitive to the inhibitory effects of warfarin.^26^ When the declining metabolic capacity with age and the increased drug needs of the overweight are factored in, there is a 40-fold variation in weekly warfarin needs. The EU-PACT trial demonstrated that genotyping at induction allowed rapid adoption of the correct dose with a more rapid achievement of a therapeutic level and greater time in range.^27^ The device used in the EU-PACT trial allowed genotyping to be done in under 2 hours without significant technical expertise and is now being evaluated in a variety of near patient settings. Other devices are under development which promise even faster and easier genotyping and dose calculation. Mega *et al*^28^ demonstrated the impact of genotyping for *CYP2C9* and *VKORC1* in a trial of edoxaban versus warfarin; in those at genotypic low risk, there was no difference in bleeding risk compared with the DOAC.

A second technical innovation has been the development of a hand-held INR testing device, the COAGuCHEK that is sufficiently simple to operate that people are able to test their INR at home and call in the result, eventually graduating in some cases to self-management of warfarin dose. A pilot study in the northeast of England has shown considerable enthusiasm among warfarin users, even those of advanced years; TTR levels of ≥80% have been recorded. In the NHS in England, the combined cost of warfarin prescription and annual INR monitoring, whether centrally organised and delivered or employing self-testing, is around £200 per annum. The annual prescription cost of the DOACs is in the range £600–800 to which should be added the cost of biannual renal function checks.

Although we have focused on anticoagulation in AF, similar considerations apply to venous thromboembolism. Projects are under way in the UK Academic Health Science Networks to deploy the combination of routine genotyping and self-testing to confirm the efficacy of more effective targeting of warfarin. Meanwhile, the NHS expenditure on anticoagulation is climbing steeply. DOACs account for 31% of treated patients but around 93% of expenditure on anticoagulants. Uptake of the new drugs varies widely, ranging from 8% to >60% in different Clinical Commissioning Groups across England^i^. As new warfarin patients decline and DOAC share of the market increases, expenditure will rise steeply despite the lack of evidence of efficacy in routine practice. Even allowing for the initial cost of genotyping of around £50, routine monitoring, typically £150 per annum which can include the depreciated £300 cost for self-testing devices on a lease arrangement, warfarin offers major savings. Warfarin costs an average of £0.83 per month, whereas the average monthly cost of DOACs is listed at just >£50. Thus, overall NHS annual expenditure could be reduced by >£0.5B per annum in the near future without impairment of the nation’s health if DOACs are restricted to those of working age and/or are shown to be sensitive to warfarin.

## Footnotes

i Primary care data are from the Business Services Authority. Secondary care data are derived from the NICE ‘Novel Oral Anticoagulant’ Technology Assessment.
