# Correction: *Direct oral anticoagulants versus warfarin: is new always better than the old?*

**DOI:** 10.1136/openhrt-2017-000712corr1

**Published:** 2018-03-01

Burn J, Pirmohamed M. Direct oral anticoagulants versus warfarin: is new always better than the old? *Open Heart* 2018;**5:**e000712. doi: 10.1136/openhrt-2017-000712

The competing interests statement was erroneously missed from this article when it first published.

­

Professor Sir John Burn is Professor of Clinical Genetics at Newcastle University and a non-executive director of NHS England where at the time of submission he was deputy chair of Specialised Commissioning at NHS England. He helped write the 2003 government White Paper on Genetics which used warfarin genotyping as an example of future deployment of Pharmacogenetics. He has been working for 10 years with a small biotechnology company called QuantuMDx Ltd, based in Newcastle, to develop point of care DNA diagnostic devices. He is now employed part time as medical director and Chair and hold shares in the company. He chose warfarin genotyping as a suitable target for development of a disposable diagnostic cassette for use in clinic. This is expected to enter trials in the next 12 months.

­

Professor Sir Munir Pirmohamed’s work on warfarin pharmacogenetics has been funded via a number of sources including Department of Health, NIHR, EU Commission and the NW Coast AHSN. The grants were awarded to the University of Liverpool. LGC, an international company, were partners in the EU-PACT Consortium, an FP7 funded EU project, and produced the point-of-care genotyping platform that was used in the EU-PACT trial. They have also provided their genotyping platform and reagents free of charge for the implementation project on warfarin pharmacogenetics which is on-going in the NW Coast (which is co-funded by the NIHR NW Coast CLAHRC and NW Coast AHSN). Professor Pirmohamed does not hold any shares in LGC or any other company marketing genotyping tests for warfarin. He also do not hold any shares in companies marketing warfarin or any of the DOACs. He is a Commissioner on Human Medicines and Chair its Pharmacovigilance Expert Advisory Group – these are independent committees which provide advice to the MHRA on the safety, efficacy and quality of medicines in the UK.

